# Underwater endoscopic submucosal dissection with dental floss traction for the treatment of early pharyngeal cancer

**DOI:** 10.1055/a-2197-9514

**Published:** 2023-11-20

**Authors:** Silin Huang, Li Tan, Suhuan Liao, Haining Cai, Guang Yang, Jianzhen Ren, Kajie He

**Affiliations:** 1 Department of Gastroenterology, South China Hospital, Medical School, Shenzhen University, Shenzhen, China; 2 Department of Gastroenterology, Beihai Peopleʼs Hospital, Beihai, China; 3 Department of Gastroenterology, Beihai People’s Hospital, Beihai, China

A 72-year-old man underwent a gastroscopy that revealed a 13 × 11-mm lesion within the left pyriform sinus (0-IIb). The lesion displayed a reddish hue under white light, with well-defined borders ( Fig. 1 a). Its tea-colored appearance under blue-laser imaging (BLI) classified it as type B1, indicative of an early pharyngeal tumor ( Fig. 1 b).

**Fig. 1:**
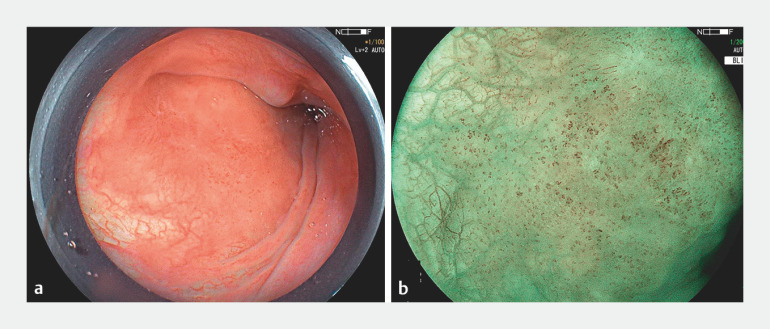
Appearance of the lesion preoperatively showing: **a** on white-light imaging, a reddish coloration of the mucosa; **b** on blue-laser imaging (BLI), a tea-colored appearance, classifying it as type B1, consistent with an early pharyngeal tumor.

Following this discovery, the patient underwent endoscopic submucosal dissection (ESD) under general anesthesia with endotracheal intubation ( Video 1 ). Magnifying endoscopy enabled precise delineation of the lesionʼs extent. Although routine procedures involving submucosal injection ( Fig. 2 a) and circumferential incision ( Fig. 2 b) were performed, challenges subsequently emerged in identifying the appropriate dissection layer owing to the confined pharyngeal space and limited submucosal thickness. To address this issue, a dental floss traction technique was employed ( Fig. 2 c), coupled with the water immersion method ( Fig. 2 d), enhancing the clarity in the submucosal dissection plane. The procedure was completed successfully, without encountering intraoperative bleeding or perforation ( Fig. 2 e,f). Subsequent pathological examination confirmed the presence of a squamous cell carcinoma, with negative resection margins. A follow-up endoscopy, 1 month post-ESD, confirmed complete healing of the surgical wound.

**Fig. 2:**
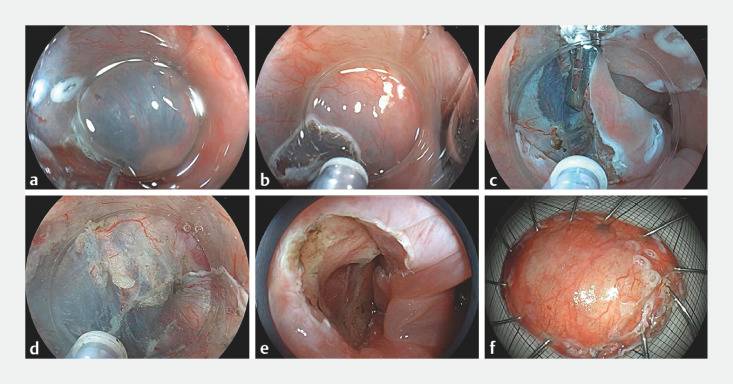
Images during underwater endoscopic submucosal dissection to treat an early pharyngeal cancer showing: **a** submucosal injection being performed; **b** a mucosal incision being made; **c** the application of dental floss traction; **d** underwater submucosal dissection being performed; **e** the postoperative appearance; **f** the macroscopic appearance of the resected tumor.

Underwater endoscopic submucosal dissection with dental floss traction is performed for a pharyngeal superficial squamous cell carcinoma.Video 1

ESD has emerged as an effective and safe therapeutic modality for early pharyngeal cancer, preserving patientsʼ quality of life and physiological function 1 2 . However, the technical complexity of the procedure is compounded by factors such as the narrow pharyngeal cavity, tracheal intubation, and the potential influence of the hyoid bone, further augmenting the challenges associated with pharyngeal endoscopy 3 .

The application of dental floss traction during ESD, along with the water immersion technique, which capitalizes on the inherent buoyancy of water, provides enhanced traction and improved visual acuity 4 . This heightened visual enhancement not only diminishes the need for injections, but also exploits waterʼs refractive amplification, thereby facilitating enhanced differentiation between the various tissue layers. Recent years have seen widespread adoption of this approach for challenging cases involving esophageal, duodenal, and colonic pathologies. To our knowledge, this case represents the first report of an early pharyngeal carcinoma treated with underwater ESD, substantiating the efficacy and safety of implementing underwater ESD in the pharyngeal region.

Endoscopy_UCTN_Code_TTT_1AO_2AC

## Notes

Endoscopy E-Videos https://eref.thieme.de/e-videos
*E-Videos* is an open access online section of the journal *Endoscopy* , reporting on interesting cases and new techniques in gastroenterological endoscopy. All papers include a high-quality video and are published with a Creative Commons CC-BY license. Endoscopy E-Videos qualify for HINARI discounts and waivers and eligibility is automatically checked during the submission process. We grant 100% waivers to articles whose corresponding authors are based in Group A countries and 50% waivers to those who are based in Group B countries as classified by Research4Life (see: https://www.research4life.org/access/eligibility/ ). This section has its own submission website at https://mc.manuscriptcentral.com/e-videos .
